# Synergistic Antitumor Effect of *Grifola frondose* Polysaccharide—Protein Complex in Combination with Cyclophosphamide in H22 Tumor-Bearing Mice

**DOI:** 10.3390/molecules28072954

**Published:** 2023-03-26

**Authors:** Jiahui Zhao, Rongjun He, Hao Zhong, Shizhu Liu, Muhammad Hussain, Peilong Sun

**Affiliations:** 1 College of Food Science and Technology, Zhejiang University of Technology, Hangzhou 310014, China; 2 Bioactives and Functional Foods Research Center, China National Light Industry, Hangzhou 310014, China; 3 Zhejiang Fangge Pharmaceutical Co., Ltd., Qingyuan 323800, China; 4 Key Laboratory of Food Macromolecular Resources Processing Technology Research, China National Light Industry, Hangzhou 310014, China

**Keywords:** polysaccharide-protein complex, *Grifola frondosa*, cyclophosphamide, hepatocellular carcinoma, tumor angiogenesis

## Abstract

Hepatocellular carcinoma (HCC) is the most common type of liver malignancy and remains a global health threat. The objective of the current study was to determine whether the combination of a cold-water extracted polysaccharide-protein complex from *Grifolia frondosa* (GFG) and cyclophosphamide (CTX) could inhibit tumor growth by suppressing the expression of angiogenesis-related proteins in H22 tumor-bearing mice. The results showed that the inhibition rate of GFG combined with CTX on H22 tumors was 65.29%, which was significantly higher than that of GFG treatment alone (24.82%). GFG combined with CTX significantly increased the expression levels of vascular endothelial growth factor, basic fibroblast growth factor, matrix metalloproteinase 2, and matrix metalloproteinase 9. Additionally, thymus index, spleen index, natural killer (NK) cell activity, interferon-γ (IFN-γ), interleukin-1β (IL-1β), tumor necrosis factor-α (TNF-α) and interleukin-2 (IL-2) levels increased significantly after GFG treatment, especially after high-doses of GFG combined with CTX treatment (*p* < 0.05). The thymus index, spleen index, NK cell activity, IFN-γ, IL-1β, TNF-α, and IL-2 levels were 1.90, 1.46, 1.30, 2.13, 1.64, 2.03, and 1.24 times of those treated with CTX alone. Thus, we proposed that GFG can alleviate the side effects of CTX by relieving the immunosuppressive effect, liver/renal injury, and oxidative stress. In conclusion, the combination of GFG and CTX for cancer treatment may be a promising strategy, and GFG is expected to be a potential adjuvant alternative for the treatment of HCC.

## 1. Introduction

Liver cancer is one of the most common malignant tumors and ranks at the top of the world morbidity and mortality rankings [1]. The high mortality rate of hepatocellular carcinoma (HCC) has accounted for 90% of primary liver cancer and made it the main form of liver cancer [2]. Currently, surgery is still the best treatment for hepatocellular carcinoma, but the prognosis for patients is poor and the recurrence rate is high [3]. In addition, some patients with advanced diseases cannot fight cancer through surgery. Therefore, conservative treatments such as chemotherapy and radiotherapy are still the first choice. Cyclophosphamide (CTX), a common chemotherapy drug, is an inactive prodrug of an alkylating agent synthesized in the 1950s [4]. CTX can be decomposed in vivo into active phosphoramide nitrogen mustard. It affects the synthesis of guanine by adding alkyl to the molecular structure of guanine, breaking the DNA double-strand structure, and eventually leading to cytotoxicity [5]. However, more and more evidence has shown that CTX has serious toxicity to normal human cells while killing tumor cells. CTX could cause bone marrow suppression and immunosuppression by influencing hematological parameters and lymphoid organs, manifested as leukopenia, lymphocyte proliferation, and cytokine reduction [6]. In addition, CTX could also produce free radicals, leading to DNA oxidative damage and oxidative stress in tissues and cells [7]. Therefore, new treatment strategies for HCC are urgently needed.

Recent studies have shown that combination therapy can effectively exert antitumor effects of CTX while reducing its side effects [8]. *Grifola frondosa* (*G. frondosa*) is a kind of edible fungus, belonging to the Aphyllophorales and Polyporaceae, which is widely distributed in Zhejiang, Hebei, and Yunnan provinces in China [9]. *G. frondosa* is rich in polysaccharides, proteins, the polysaccharide-protein complex (PPC), vitamins, and a variety of trace elements, which have a variety of biological activities such as immune regulation, anti-tumor activity and anti-oxidation [10]. *G. frondosa* PPC is a polymer consisting of polysaccharides and proteins/peptides linked by covalent bonds. The structure and biological activity of PPCs are different from polysaccharides, which could activate innate immunity and adaptive immunity [11]. In our previous studies, it has been demonstrated that *G. frondosa* PPC has anti-proliferation effects on hepatocytes in vitro [12]. Reports of *Coriolus versicolor* PPC bioactivity are common in the literature. Chan et al. showed that the *Coriolus versicolor* peptide enhanced CTX toxicity to HepG2 cells, which decreased HepG2 cell viability by 22% compared with CTX alone [13]. Another study demonstrated that *Coriolus versicolor* peptide restored CTX-induced lymphocyte proliferation inhibition, natural killer cell function, white blood cell production, and spleen and thymus growth in rats [14]. Studies on the anti-tumor effects of *G. frondosa* polysaccharide combined with CTX have also been reported. Guo et al. found that *G. frondosa* polysaccharide eliminated CTX-induced decreases in interleukin-2 (IL-2), interleukin-6 (IL-6), and tumor necrosis factor-α (TNF-α) levels. In addition, *G. frondosa* polysaccharide increased the natural killer (NK) cytotoxicity, lymphocyte proliferative activity, and the activities of antioxidant enzymes such as total superoxide dismutase (T-SOD), catalase, and glutathione peroxidase (GSH-Px) in immunosuppressed mice [15]. Nie et al. proposed that sulfated polysaccharide derived from *G. frondosa* could accelerate the anti-Sarcoma 180 (S180) tumor activity of CTX and improve the immune response to CTX injury [16]. All the above studies indicated that *G. frondosa* polysaccharide combined with CTX may increase the toxicity of cancer cells and reduce the toxicity and side effects of CTX in cancer therapy. Certainly, in addition to *G. frondosa* polysaccharide, the reports of anti-tumor effects of other edible fungus polysaccharides combined with CTX remain more numerous. Cui et al. found that the combination of *Dioscorea bulbifera* polysaccharide and CTX could potentially enhance the antitumor effect of CTX and reduce CTX-induced immunosuppression and oxidative stress in U14 cervical tumor-bearing mice [17]. The inhibitory rate of *Boletus aereus* polysaccharide combined with CTX on the S180 tumor was 63.32%, which was significantly higher than that of CTX or *Boletus aereus* polysaccharide alone (*p* < 0.05) [18]. Li et al. proposed that *Ganoderma atrum* polysaccharide used in combination with CTX was superior to either one alone [19]. Additionally, some plant or animal polysaccharides can also improve the therapeutic effectiveness of CTX. Zong et al. found that a heteropolysaccharide obtained from the ink of the cuttlefish, *Sepiella maindroni* de Rochebruns, combined with treatment with CTX showed a higher anti-S180 activity [20]. A polysaccharide isolated from *Schisandra chinensis* (Turcz.) Baill increased the thymus and spleen indexes and the activity of peritoneal macrophages and spleen cells, alleviating immunosuppression induced by CTX [21]. When ginseng is combined with certain anticancer drugs, the anti-tumor effect will be enhanced [22]. Overall, combination therapy can be better used for the treatment of liver cancer by reducing the toxicity of chemotherapy drugs and improving the antitumor activity.

The treatment of tumors depends on the host immune system, and the immunomodulatory effects of *G. frondosa* polysaccharide coincidentally compensate for the immunosuppression induced by CTX in tumor therapy. Based on the above conclusions, we speculated that the combination of *G. frondosa* PPC and CTX could also exert better anti-hepatocellular carcinoma activity and alleviate the side effects induced by CTX simultaneously. Hence, this research investigated the anti-tumor effect of GFG (*G. frondosa* PPC was extracted from *G. frondosa* at 4 °C) in combination with CTX on H22 tumor-bearing mice and its underlying molecular mechanism. In addition, the mitigation potentials of GFG on the side effects caused by CTX were also evaluated, which provided a basis for the synergistic treatment of cancer with active components of natural edible fungi and chemical drugs.

## 2. Results

### 2.1. GFG Combine Treatment with CTX Enhanced the Anti-Tumor Effect of H22 Tumor-Bearing Mice

**Figure 1:**
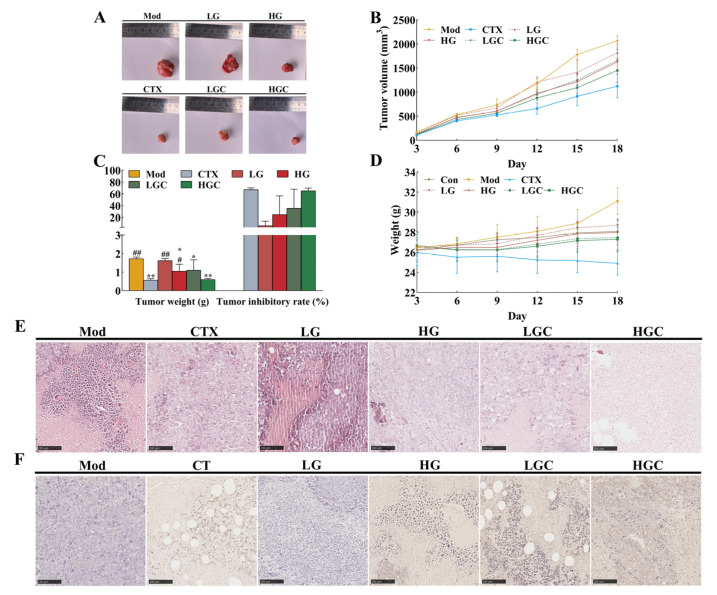
The inhibition effect of GFG combined with CTX on the H22 tumor-bearing mice. (**A**) Representative photos of tumors in the various groups. (**B**) Changes in tumor volume induced by different treatments. (**C**) Changes in tumor weight and tumor inhibition rate in the various groups. (**D**) Changes in body weight of mice in the various groups. (**E**) H&E staining and (**F**) TUNEL staining of the tumor samples in different groups. Data were expressed as the mean ± SD. * *p* < 0.05 and ** *p* < 0.01 versus the Mod group; # *p* < 0.05 and ## *p* < 0.01 versus the CTX group.

Obviously, comparing the size of tumor tissues among different groups, it was found that the huge reductions in tumor volume could be visually seen in the CTX, HG, LGC, and HGC groups, especially in the HGC group (Figure 1A). The line plots and histograms in Figure 1B,C depict the changes in tumor volume and weight. Compared with the Mod group (1.72 g), the tumor weights of the HG group, LGC group, and HGC group were 1.05 g, 1.10 g, and 0.60 g, respectively, showing significant differences (*p* < 0.05). In addition, the tumor inhibition rate of mice treated with high-dose GFG and CTX was up to 65.29%, significantly higher than that of mice treated with high-dose GFG alone (*p* < 0.05). These results indicated that both a certain dose of GFG or CTX alone and the combination treatment had an anti-tumor effect. Figure 1D exhibited the changes in body weight of the different groups of mice. Obviously, the Mod group had the largest body weight, and the CTX group had the most negligible body weight. The former might be caused by tumor progression, while the latter might be due to the cytotoxicity of CTX, which inhibited tumor and body weight growth. Notably, the LGC and HGC groups showed a significant increase in body weight compared with the CTX group and were close to the Con group, implying that GFG could alleviate the cytotoxicity of CTX.

### 2.2. Morphological Changes of Tumor Tissues

To further verify the anti-tumor effect of GFG combined with CTX in vivo, this study used H&E staining and TUNEL staining for pathological analysis of tumor tissues. As shown in Figure 1E, the tumor cells in the Mod group were arranged neatly, and the nuclei were clearly visible, which showed obvious morphological characteristics, indicating that the tumor cells were in a proliferative state. In contrast, different degrees of tumor necrosis and loose cell arrangement occurred in the treatment groups, especially in the HGC group. This phenomenon indicated that a high dose of GFG combined with CTX had obvious toxicity to tumor cells. Consistently, compared with the Mod group, the CTX, HG, LGC, and HGC groups had obvious apoptotic areas (brown areas) in TUNEL staining (Figure 1F). As expected, the numbers of apoptosis in the LGC and HGC groups were significantly higher compared with the CTX group (Figure 1F), indicating that the combination of GFG and CTX had a stronger apoptosis-inducing effect in the treatment of H22 tumor cells.

### 2.3. The Combination of GFG and CTX Enhanced Immune Response of H22 Tumor-Bearing Mice

**Figure 2:**
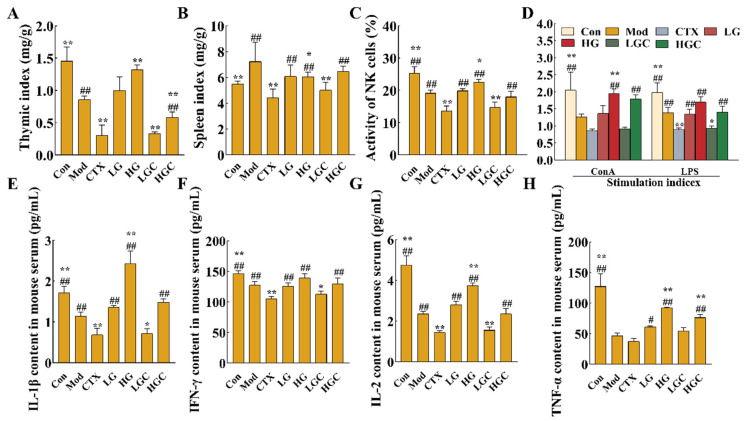
The immunomodulatory effects of GFG combined with CTX on H22 tumor-bearing mice. (**A**,**B**) Thymus and spleen index. (**C**,**D**) NK cell killing activity and proliferation of splenic lymphocytes. (**E**–**H**) Effects of GFG combined with CTX on serum IFN-γ, IL-1β, TNF-α and IL-2 levels in H22 tumor-bearing mice. Data were expressed as the mean ± SD. * *p* < 0.05 and ** *p* < 0.01 versus the Mod group; # *p* < 0.05 and ## *p* < 0.01 versus the CTX group.

In the process of growth, tumor cells can escape the surveillance of the immune system by recruiting immunosuppressive cells or molecules, modifying their own surface antigens, and changing the tumor microenvironment, and thus immune escape occurs, leading to tumor progression. The activation of immune cells and secretion of cytokines can activate the immune system and promote immune regulation to effectively kill tumor cells [23]. Therefore, this study evaluated the effects of GFG combined with CTX on immune organs, immune cells, and cytokines in H22 tumor-bearing mice. As shown in Figure 2A,B, the thymus index of mice treated with high doses of GFG combined with CTX was significantly lower than that of the Mod group and significantly higher than that of the CTX group (*p* < 0.05). Additionally, GFG alone treatments significantly increased the thymus index of H22 tumor-bearing mice (*p* < 0.01). These results indicated that a high dose of GFG could enhance the immune function of H22 tumor-bearing mice and restore the impaired immunomodulatory effect induced by CTX. Similarly, CTX reduced the splenic index, whereas GFG alone or combined with CTX could reverse this effect. Consequently, the side effects of CTX on immune organs could be alleviated by GFG combination therapy.

NK cells and lymphocytes play an indispensable role in tumor recognition and elimination. Among them, NK cells exert cytotoxicity by producing antigen-independent immune responses against malignant cells. Figure 2C shows the detection results of NK cell activity in H22 tumor-bearing mice. The activity of NK cells in the Mod group was significantly lower than that in the Con group, indicating that the ectopic xenograft tumor growth inhibited mice’s immune function (*p* < 0.01). The NK cell activity of H22 tumor-bearing mice was significantly increased after oral administration of 300 mg/kg GFG (*p* < 0.05). CTX, an immunosuppressant used in cancer treatment, causes severe immunosuppression. Thus, the NK cell activity of mice injected with CTX alone was significantly lower than that of Mod group (*p* < 0.01). However, high-dose GFG combined with CTX significantly reversed the trend of NK cell activity in the CTX group (*p* < 0.01). Altogether, these results indicated that GFG could alleviate the immunosuppression induced by CTX.

The proliferation activity of splenic lymphocytes could also assess cellular immune function to a certain extent. T lymphocytes are mainly responsible for cellular immunity, while B lymphocytes exert humoral immunity by producing antibodies. Figure 2D showed the proliferative capacity of splenic lymphocytes (concanavalin A (Con A)-stimulated T cells and lipopolysaccharide (LPS) stimulated B cells) in each group. It was observed that high-doses of GFG markedly reversed the immunosuppression induced by the malignant growth of H22 tumors. Compared with the CTX group, high-dose GFG combined with CTX significantly increased the proliferation of T and B lymphocytes (*p* < 0.01). These results indicated that high doses of GFG could activate the immune response in tumors and exert immunomodulatory effects. In addition, the results again confirmed that the combination of GFG and CTX could alleviate the side effects caused by CTX.

CTX could inhibit the secretion of cytokines, thus inhibiting the immune activity of mice. The levels of cytokines in serum were shown in Figure 2E–H. After CTX treatment, the levels of IFN-γ, interleukin-1β (IL-1β), IL-2 and TNF-α in the serum decreased to 104.98 pg/mL, 0.69 pg/mL, 1.44 pg/mL, and 37.64 pg/mL, respectively. These results suggested that CTX had an immunosuppressive effect on the immune system of mice. The levels of cytokines in the treatment group with different doses of GFG were significantly increased, which indicated that GFG had the effect of activating the immune system. The serum cytokine content in the high-dose GFG and CTX combined treatment group was significantly higher than that in the CTX alone treatment group (*p* < 0.01), proving that GFG could alleviate the immunosuppressive effect of CTX. Conclusively, GFG could activate and regulate the immune system by increasing cytokine levels.

**Figure 3:**
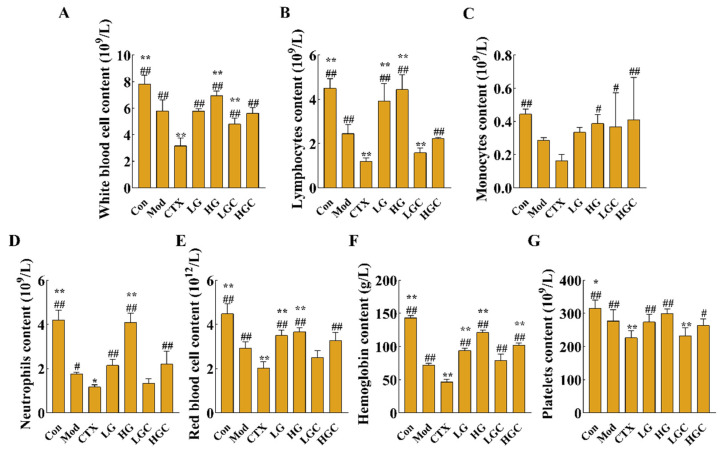
The blood routine examination in each group of mice. (**A**) The concentration of white blood cells in different groups. (**B**) The concentration of lymphocytes in different groups. (**C**) The concentration of monocytes in different groups. (**D**) The concentration of neutrophils in different groups. (**E**) The concentration of red blood cells in different groups. (**F**) The concentration of hemoglobin in different groups. (**G**) The concentration of platelets in different groups. Data were expressed as the mean ± SD (*n* = 8). * *p* < 0.05 and ** *p* < 0.01 versus the Mod group; # *p* < 0.05 and ## *p* < 0.01 versus the CTX group.

Hematological toxicity assessment results are shown in Figure 3. Compared with the Mod group, CTX treatment alone markedly decreased the concentrations of white blood cells (WBC), lymphocytes (Lymph), monocytes (Mon), neutrophils (Gran), red blood cells (RBC), platelets (PLT) and hemoglobin (HGB) (*p* < 0.05). After GFG intervention, the number of these immune cells was 1.78, 1.85, 2.56, 1.87, 1.62, 2.18, or 1.16 times greater than that of the CTX alone treatment group (*p* < 0.05). Collectively, these data proved that GFG could alleviate CTX-induced immunosuppression.

### 2.4. The Combination of GFG and CTX Reduced the Expression of Angiogenesis-Related Proteins in Tumor Tissues

**Figure 4:**
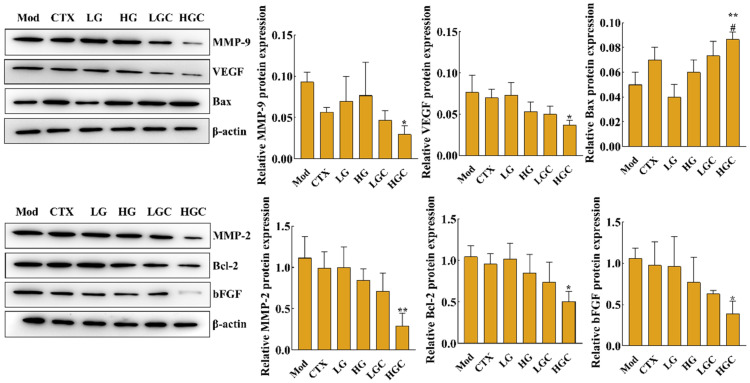
Effect of GFG combined with CTX on the expression of MMP-9, MMP-2, VEGF, bFGF, Bcl-2 and Bax proteins in tumor tissue analyzed by western blotting. Data were expressed as the mean ± SD. * *p* < 0.05 and ** *p* < 0.01 versus the Mod group; # *p* < 0.05 versus the CTX group.

Studies have shown that the expression level of vascular endothelial growth factor (VEGF) is closely related to the development of malignant tumors [24]. To confirm whether the combination of GFG and CTX inhibits the H22 tumor growth by affecting the expression of angiogenesis-related proteins, the protein levels of matrix metalloproteinase-9 (MMP-9), metalloproteinase-2 (MMP-2), VEGF, basic fibroblast growth factor (bFGF), B-cell lymphoma-2 (Bcl-2) and Bcl-2 assayed X protein (Bax) were determined by western blotting. Compared with the Mod group, a high-dose of GFG combined with CTX significantly reversed the protein levels of MMP-9, MMP-2, VEGF, bFGF, Bcl-2, and Bax (Figure 4, *p* < 0.05). In addition, MMP-9, MMP-2, VEGF, bFGF, and Bcl-2/Bax were down-regulated by CTX or GFG treatment, but not significantly. These results suggested that the combination treatment with GFG and CTX exerted an anti-hepatocellular carcinoma role by inhibiting angiogenesis regulators in tumors.

### 2.5. GFG Alleviated the Liver Injury and Oxidative Stress Induced by CTX

**Figure 5:**
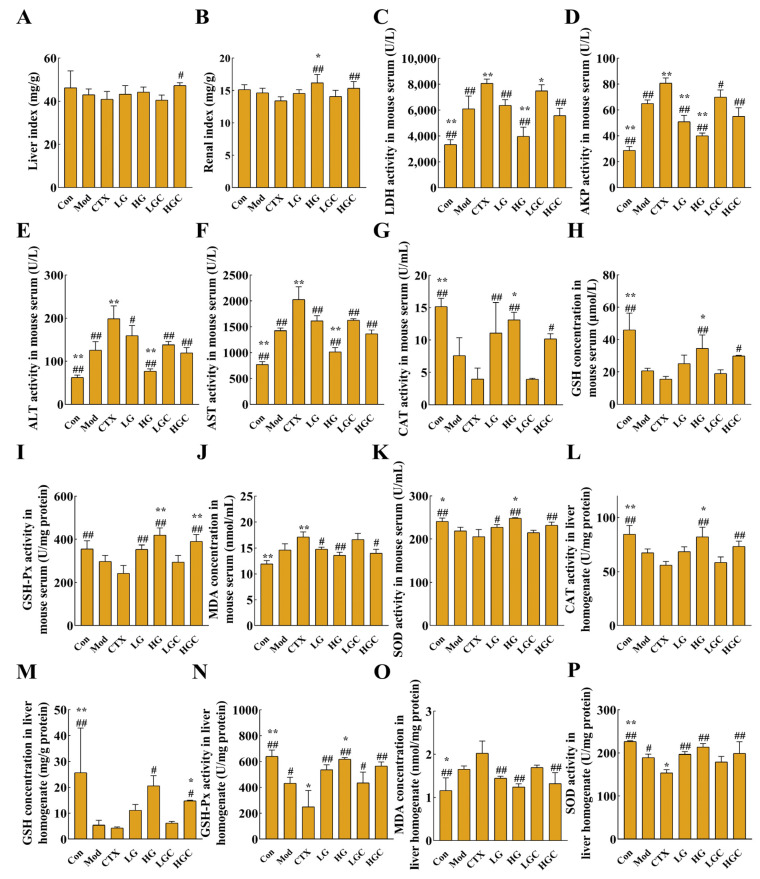
GFG relieved the side effects induced by CTX. (**A**,**B**) Liver and renal indexes of mice in different groups. (**C**–**F**) Changes of liver damage indicator. (**G**–**K**) Changes of oxidative damage indicators (the activities of GSH-Px, CAT, SOD and contents of MDA and reduced GSH in serum) in serum. (**L**–**P**) Changes of liver damage indicator (the activities of GSH-Px, CAT, SOD and contents of MDA and reduced GSH in liver tissues) in liver tissues. Data were expressed as the mean ± SD. * *p* < 0.05 and ** *p* < 0.01 versus the Mod group; # *p* < 0.05 and ## *p* < 0.01 versus the CTX group.

To test the hypothesis that GFG may alleviate the metabolic tissue injury of CTX on immune organs, liver and kidney indices were analyzed (Figure 5A,B). As important organs of drug metabolism, the liver and kidney could reflect drug toxicity. High-dose combination therapy of GFG and CTX significantly alleviated the decrease in organ index after CTX treatment alone, suggesting that GFG may ameliorate CTX-induced liver and renal injury.

The activities of alkaline phosphatase (AKP), alanine aminotransferase (ALT), lactate dehydrogenase (LDH), and aspartate aminotransferase (AST) are important indicators to reflect the degree of liver injury [25]. When the liver is damaged, the permeability of the liver cell membrane increases, thereby allowing the release of AKP, ALT, LDH, and AST from the cells into the serum and resulting in increased enzymatic activity of these enzymes in the serum. In this study, the activities of these enzymes in serum were detected, and the results are shown in Figure 5C–F. Compared with healthy mice, the activities of AKP, ALT, LDH, and AST were significantly increased in the Mod group, indicating that H22 tumor formation could cause liver injury in mice. However, GFG decreased the activities of the four enzymes in a dose-dependent manner, suggesting that GFG had a protective effect on the liver. Similarly, compared with the CTX group, the combined treatment group significantly decreased the activities of AKP, ALT, LDH, and AST in a dose-dependent manner, proving that GFG alleviated CTX-induced liver injury.

Another common side effect of CTX is oxidative stress, which severely limits the application of CTX. To evaluate the state of oxidative stress, the activities of glutathione peroxidase (GSH-Px), CAT, SOD, and the contents of malondialdehyde (MDA) and reduced glutathione (GSH) in serum and liver tissue were determined in this study. As shown in Figure 5G–P, compared with the Con group, the content of MDA in the CTX group and Mod group showed an upward trend, while other indexes showed a downward trend in serum and liver tissue. MDA is a product of externally induced lipid peroxidation and a carbon-based compound produced by oxidative stress. Overproduction of MDA will aggravate tissue damage. After treatment with different doses of GFG combined with CTX, the activities of GSH-Px, CAT, SOD, and the contents of MDA, as well as reduced GSH in serum and liver tissue, were reversed to different degrees, especially in HGC group (*p* < 0.05). These results indicated that GFG could ameliorate the oxidative stress caused by the H22 ectopic xenotransplantation and CTX.

### 2.6. H&E Staining Analysis of Liver and Kidney Tissues

**Figure 6:**
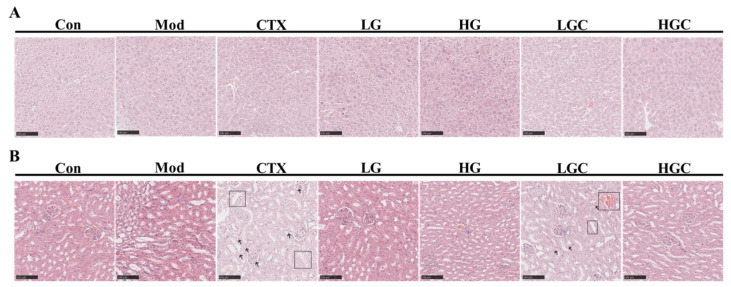
Histological analysis of liver and kidney. (**A**) H&E staining analysis of liver tissues. (**B**) H&E staining analysis of renal tissues in different groups of mice. Scale bar, 100 μm.

Histological examination of the liver and kidney was displayed in Figure 6A,B. Hepatocytes in the Con group were arranged neatly and in a regular shape, with good growth status. However, hepatocytes in the CTX group showed vacuolated and loose cytoplasm with blurred boundaries. Interestingly, the necrosis of these cells improved after GFG treatment. In the CTX group, renal tubule dilation and lumen obstruction were clearly observed under the microscope, and multiple neutrophils were infiltrated. Similar features were partially presented in the LGC group, but these pathological features were recovered in the HGC group. In addition, no such phenomenon was observed in groups treated with different doses of GFG. These results suggested that a moderate dose of GFG had no adverse effects on the liver and kidney and could alleviate CTX-induced liver and kidney damage.

## 3. Discussion

HCC is a common primary liver cancer with a high mortality rate worldwide. However, current treatments remain many problems such as poor prognosis and severe-side effects [26]. CTX is a broad-spectrum cytotoxic drug that could kill tumor cells. Nevertheless, CTX could also non-specifically kill immune cells and induce DNA damage in normal cells, which adversely affects quality of life for many HCC survivors [27]. *Grifola frondosa*, a large edible fungus, has been widely used in food and health products because of its anti-tumor and immunomodulatory activities. In our previous report, GFG with a molecular weight of 2190 kDa was shown to inhibit hepatocyte proliferation through mitochondrial apoptosis and the Fas/FasL pathway [12]. This study mainly investigated the effect of GFG combined with CTX in the treatment of hepatocellular carcinoma in vivo.

H22 tumor-bearing mice were used to investigate the effects of the combination treatment with GFG and CTX on anti-tumor effects and reducing side effects of CTX in our experiment. The results showed that CTX alone could effectively inhibit H22 tumor growth compared with the Mod group (*p* < 0.01, Figure 1A,B). However, mice in the CTX group began to lose weight at day 9 and decreased to 24.90 g at day 18. These results are consistent with those reported by Yang et al. [28], which found that after 3 days of intraperitoneal injection of 80 mg/kg CTX, the weight gain of mice was significantly decreased compared with the blank group. Above results indicated that weight suppression is a vital indicator for CTX toxicity. The immune system is an important defense system that has the functions of monitoring, defense, and regulation; those functions are closely related to the occurrence and development of tumors. The whole immune system consists of immune organs, immune cells, and immune molecules [29]. Thymus and spleen are important lymphoid organs and immune organs of the body [30]. Thymus index and spleen index can well reflect the immunotoxicity of drugs and the body’s immune state [31]. The results suggested that treatment with GFG alone or in combination with CTX could significantly alleviate the dysfunction of immune organs caused by xenografts (*p* < 0.05). Particularly, the effect of GFG combined with CTX was obviously higher than that of GFG alone. In addition to the fact that the occurrence of tumors can inhibit immune function, the application of chemotherapy drugs will further aggravate the immune suppression. Therefore, it is of great significance to improve the immune function, especially to improve the state of severe immunosuppression during chemotherapy. Our results showed that the addition of GFG evidently alleviated the cytotoxicity of CTX to the thymus and spleen. A similar study reported by Cao et al., which suggested that a sulfated polysaccharide from the green alga *Ulva conglobata* Kjellman could elevate the spleen and thymus indexes improve the damage of cyclophosphamide to spleen and thymus [32]. Liu et al. showed that 375 mg/kg *Panaxnotoginseng* polysaccharide markedly reversed the decrease in thymus index caused by CTX [8]. Therefore, we proposed that GFG combined with CTX could effectively reduce the toxicity of immune organs in CTX-induced H22 tumor-bearing mice to achieve better antitumor effects than CTX treatment. Immune cells such as NK cells and T lymphocytes can specifically recognize tumors and effectively impede their growth [33]. It has been reported that the proliferation of NK cells can promote a good prognosis for solid tumors [34]. The treatment with GFG alone or in combination with CTX significantly increased the activities of NK cells and T lymphocytes in the Mod group and CTX group (*p* < 0.05). It is reported that the decrease of NK cells would increase the invasion and metastasis of cancer cells [35], which supported the finding that GFG significantly restored the decreased activity of immune cells in H22 tumor-bearing mice caused by CTX. Zhai et al. also indicated that chitosan oligosaccharides enhanced the cytotoxicity of NK cells in S180 residual-tumor mice [36]. In addition, immune molecules such as TNF-α, INF-γ, IL-1β, and IL-2 were also determined in the present study. The results showed that the combination of GFG and CTX activated the host immune system and alleviated CTX-induced immunosuppression. Wang et al. observed a similar tendency for changes in cytokines as in our study when they studied the extracts from Yifei Tongluo Granules. It was suggested that the extracts from Yifei Tongluo Granules could promote the secretion of cytokines, reducing the severity of immunosuppression [37].

Angiogenesis is a biological behavior of tumor growth and plays an important role in tumor growth and migration [38]. Currently, anti-tumor angiogenesis has become one of the important targets of anti-tumor drugs. It has been shown that polysaccharides can play a vital role in antitumor therapy by suppressing the formation of blood vessels. Zhao et al. showed that *Ulva lactuca* polysaccharide directly kills tumor cells and suppresses tumor proliferation by inhibiting angiogenesis [39]. Wang et al. demonstrated that ethanol extract from *Pholiota adiposa* exhibited antitumor activity in vivo by promoting apoptosis and inhibiting angiogenesis [40]. Qiu et al. proposed that *Gastrodia elata* polysaccharide could inhibit the BMP/Smad/Id1 signaling pathway and block the formation of new angiogenesis, thus suppressing the growth of hepatocellular carcinoma in mice [41]. Angiogenesis is the process by which endothelial cells from existing blood vessels infiltrate the tumor to form new blood vessels. Endothelial cells are the main cellular components of new blood vessels and are mainly regulated by VEGF. VEGF is an important factor regulating the growth of blood vessels, which promotes angiogenesis and participates in the process of tumor growth by binding to specific receptors on the surface of vascular endothelial cells [42]. In addition, angiogenin, transforming growth factor (TGF-β), and bFGF are also important factors promoting the maturation of new capillaries. Among them, bFGF binds to a receptor on the target cell, and the receptor dimerization further activates tyrosine kinase and intracytoplasmic protein phosphorylation, initiating a signaling cascade [43]. Finally, it causes cell proliferation and differentiation and promotes tissue regeneration and angiogenesis. Additionally, matrix metalloproteinases can control tumor neovascularization, regulate cell adhesion and movement, and regulate tumor cell growth by influencing intracellular signals, which directly or indirectly participate in various physiological and pathological processes. MMP-2 can promote the transformation of the angiogenic phenotype, and MMP-9 can activate the generation of blood vessels, which is the prerequisite for endothelial cells to form new blood vessels [44]. Western blot was performed to detect and analyze the expression of angiogenesis-related proteins in tumor tissues of each group of mice in the present study. Compared with the CTX group, the expression levels of MMP-9, MMP-2, VEGF, bFGF, Bax, and Bcl-2 in tumor tissues were reversed by 47%, 72%, 49%, 60%, 57%, and 48%, respectively, after high-dose GFG combined with CTX treatment. These results indicated that combined treatment had a significantly better inhibitory effect on tumor angiogenesis than CTX alone, which may be a potential mechanism to inhibit tumor growth. Studies have shown that immune factors such as TNF and IFN can not only directly kill tumor cells but also restrain the formation of blood vessels, thus inhibiting tumor growth [45]. Current results showed that CTX significantly reduced TNF-α and IFN-γ levels in serum, while GFG treatment mitigated this inhibition. This suggested that the immunosuppressive effect of CTX may lead to tumor resistance mediated by angiogenesis and that the intervention of GFG compensates for the absence of CTX on angiogenesis. Therefore, combined treatment has a stronger antitumor effect than treatment with CTX alone. One study reported that hypoxia induces the expression of multiple hypoxia-inducible factor-1α (HIF-1α) target genes in most cell types, and constitutionally activated signal transducer and activator of transcription 3 (Stat3) can directly up-regulate the expression of VEGF and HIF-1α [46]. Therefore, GFG may inhibit the production of VEGF by inhibiting the activation and expression of Stat3 and its effector factor HIF-1α. Additionally, Wilken et al. proved that nuclear factor kappa-B (NF-κB) can directly promote the activation and expression of angiogenic genes [47]. Unfortunately, present study ignored the effect of GFG on the expression of NF-κB in tumor tissues. We speculate that this may also contribute to GFG’s angiogenesis inhibition. Conclusively, all above results suggested that combined treatment with CTX and GFG can play an essential role in antitumor therapy by suppressing angiogenesis.

## 4. Materials and Methods

### 4.1. Materials and Reagents

H22 cells were purchased from the Beijing Cell Bank of the Chinese Academy of Sciences (Beijing, China). CTX was purchased from Aladdin Reagent Co., Ltd. (Shanghai, China). GFG were prepared as described in our previous work [12]. In brief, the degreased *G. frondosa* fruiting body powder was extracted by cold water (*w*/*v* = 1:20, 4 °C, 20 min, 3 times). The supernatant was passed through a tubular ceramic membrane (MW cutoff, ≥500 kDa; Anhui, China). Afterward, the condensed trapped solution was further purified by a DEAE Sepharose Fast Flow column (60.6 × 218.5 mm) and an XK 26/100 Sephacryl S-300 HR column (26 × 1000 mm) to obtain GFG. The total sugar content and the protein content of GFG were 21.20% and 30.69%, respectively. The molecular weight of GFG was 2190 kDa. The main monosaccharides of GFG were rhamnose, glucose, and galactose, with an approximate ratio of 3.00: 1.00: 0.86. Fetal bovine serum (FBS), penicillin-streptomycin solution, and RPMI-1640 were acquired from Thermo Fisher Scientific Co., Ltd. (Shanghai, China). Reduced GSH, GSH-Px, LDH, MDA, T-SOD, total protein (TP), and TUNEL apoptosis detection kits were all provided by Nanjing Jiancheng Bioengineering Institute (Nanjing, China). ELISA kits for IL-1β, IL-2, IFN-γ and TNF-α were purchased from Multi Sciences (Lianke) Biotech Co., Ltd. (Hangzhou, China).

### 4.2. Cell Culture and Animal Model Design

H22 cells were cultivated in RPMI-1640 medium supplemented with 10% FBS and 1% penicillin-streptomycin. H22 cells were incubated at 37 °C under 5% CO_2_.

Male BALB/c mice (6 weeks, 20 ± 2 g) were obtained from Beijing Vital River Laboratory Animal Technology Co., Ltd. (Beijing, China) (qualified number: 20220527Abzz0619080528). Animals were housed with sufficient food and water available ad libitum for 7 days in standard animal rooms, with humidity at 50–55%, temperature of 20 ± 25 °C and 12 h light/dark cycle. All experimental procedures in our study were approved by the Animal Ethics Committee of Zhejiang Chinese Medical University, and the animal ethics approval number was 20220613-12.

BALB/c mice (6 weeks, 20 ± 2 g) were randomly divided into seven groups (*n* = 8 each): the control group (Con), the model group (Mod), the positive control group (CTX), the 100 mg/kg GFG group (LG), the 300 mg/kg GFG group (HG), the 100 mg/kg GFG + 25 mg/kg CTX group (LGC), and the 300 mg/kg GFG + 25 mg/kg CTX group (HGC).

H22 cell suspension (0.2 mL) at a concentration of 2 × 10^6^ cells/mL was injected peritoneally into three mice for cell proliferation. One week later, mouse ascites was collected and prepared with sterile saline to form a 2 × 10^6^ cells/mL cell suspension, which was then subcutaneously inoculated with 0.2 mL of the cell suspension into the right armpit of mice. After 3 days of feeding, the mice were treated with drug administration, as shown in Table 1. During the 21 days of treatment, body weight and tumor sizes were monitored every 3 days. Tumor volume was calculated using the formula: tumor volume (mm^3^) = LW^2^/2 (L, tumor length; W, tumor width). After blood collection, BALB/c mice were sacrificed via neck dislocation. Tumor, liver, spleen, kidney and thymus were removed for further analysis. The inhibition rate of tumor growth was calculated as follows: inhibition rate (%) = (1—tumor volume of test group/tumor volume of Con group) × 100%. The spleen index and thymus index were calculated using the following equation: spleen (thymus) index (g/g) = (spleen or thymus mass/total weight) × 100%.

### 4.3. Biochemical Determination

The biochemical indicators and inflammatory factor level in serum and tissue were determined using the enzyme-linked method described by Zong et al. [48]. The whole blood was collected and analyzed by an automatic hematology analyzer. Serum was obtained by centrifuging the blood at 1000× *g* for 10 min at 4 °C. The serum levels of AKP, ALT, LDH, and AST were measured by an automatic biochemical analyzer (Sysmex, Japan). To prepare 10 % (*w*/*v*) tissue homogenates, 200 mg of liver tissue were homogenized in 2.8 mL of sterile normal saline (4 °C) and centrifuged at 1000× *g* for 5 min. The activities of GSH-Px, CAT, and T-SOD and the concentrations of MDA and reduced GSH in the liver and serum were tested by kits. The levels of IFN-γ, IL-1β, TNF-α, and IL-2 in serum were measured using a mouse enzyme-linked immunosorbent assay according to the manufacturer’s protocols.

### 4.4. H&E Staining and TUNEL Assays

Tumor tissues were fixed in 4% paraformaldehyde for 24 h, then embedded in paraffin and sectioned. Tissues slides were stained using the H&E staining kit according to the manufacturer instructions. Afterward, changes in the tissue structure were observed under a light microscope at ×20 magnification.

A TUNEL assay kit was used to detect tumor apoptosis according to the manufacturer’s instructions. Tissue sections were incubated with proteinase K solution for 15 min and incubated in 3% hydrogen peroxide for 20 min prior to adding 50 μL TUNEL solution at 37 °C for 1 h. Images were observed at ×20 magnification using a fluorescence light microscope.

### 4.5. Natural Killer Cell Activity

Splenocytes were harvested for NK cell activity analysis. Spleen cells (1 × 10^6^ cells/well) were incubated with YAC-1 cells (4 × 10^4^ cells/well) at a 5: 1 effector: target ratio for 4 h at 37 °C. The resultant cell suspension was then centrifuged at 250× *g* for 5 min before taking the supernatant for analysis. Absorbance was detected at 492 nm using a microplate reader (Bio-Rad, Hercules, CA, USA).

### 4.6. Spleen Lymphocyte Proliferation Assay

To activate T or B cells, Con A and LPS were used to treat splenocytes. Spleen-cell suspensions containing 5 × 10^6^ cells/mL were added to Con A at a final concentration of 5 μg/mL and LPS at a final concentration of 10 μg/mL with RPMI-1640 medium containing 10% FBS. After 72 h of incubation at 37 °C, the supernatant was removed and 200 μL of dimethyl sulfoxide was added. Finally, the absorbance was measured at 570 nm using a microplate reader (Bio-Rad, Hercules, CA, USA).

### 4.7. Statistical Analysis

Values were expressed as means ± standard deviation (SD) and analyzed by one-way ANOVA with a statistical analysis software package (GraphPad Software, San Diego, CA, USA). *p* < 0.05 was considered significantly different, while *p* < 0.01 was considered extremely significant.

## 5. Conclusions

In conclusion, GFG and CTX exhibited anti-tumor effects by synergistically inhibiting H22 tumor angiogenesis. Compared with the Mod group, the expression levels of MMP-9, MMP-2, VEGF, bFGF, Bax, and Bcl-2 in tumor tissues were reversed by 67%, 74%, 50%, 63%, 80%, and 51%, respectively, after GFG combined with CTX treatment (*p* < 0.05). Additionally, GFG could alleviate the liver injury and oxidative stress of CTX and promote the secretion of immune cytokines, activating the host immune system. Therefore, the current study provided a practical basis for GFG as an ancillary drug to combine therapy with CTX, which may also become a more effective anti-hepatocellular carcinoma strategy in the future. Notably, as a common anti-tumor chemotherapy drug, CTX can destroy the intestinal barrier of chemotherapy patients and cause an imbalance of gut microbiota in addition to immunosuppression and other side effects [49]. Edible mushroom polysaccharide, or PPC, can regulate the composition of gut microbiota and maintain gut homeostasis. Therefore, whether edible mushroom polysaccharide or PPC could alleviate the negative effects of CTX on gut microbiota can be regarded as the direction of future research so as to fully explore the biological activities of edible mushrooms in tumor therapy, immune activation, and gut microbiota regulation.

## Figures and Tables

**Table 1:** Experiment grouping and treatment methods.

Group	Con	Mod	CTX	LG	HG	LGC	HGC
Sterile water (mL)	0.2 ^a^, 0.2 ^b^	0.2 ^a^, 0.2 ^b^	0.2 ^a^	0.2 ^b^	0.2 ^b^		
100 mg/kg GFG (mL)				0.2 ^a^		0.2 ^a^	
300 mg/kg GFG (mL)					0.2 ^a^		0.2 ^a^
25 mg/kg CTX (mL)			0.2 ^b^			0.2 ^b^	0.2 ^b^

Note: 0.2 ^a^, oral gavage, 0.2 mL; 0.2 ^b^, intraperitoneal inject 0.2 mL. Con, normal control group; Mod, model control group; CTX, cyclophosphamide (CTX) positive control group; LG, 100 mg/kg GFG group; HG, 300 mg/kg GFG group; LGC, 100 mg/kg GFG + 25 mg/kg CTX group; HGC, 300 mg/kg GFG + 25 mg/kg CTX group.

## Data Availability

Not applicable.
